# Dynamic changes of platelets before and after surgery predict the prognosis of patients with operable non-small cell lung cancer

**DOI:** 10.7150/jca.65129

**Published:** 2022-01-01

**Authors:** Wei Yang, Yating Yao, Yingying Chen, Fangsi Jin, Tingting Zheng, Xinyi Ai, Ting Zhang, Chunming Ding, Minghua Jiang

**Affiliations:** 1 School of Laboratory Medicine and Life Science, Wenzhou Medical University, 325035 Wenzhou, Zhejiang, China.; 2 Key Laboratory of Laboratory Medicine, Ministry of Education, Wenzhou Medical University, 325035 Wenzhou, Zhejiang, China.; 3 Department of Laboratory Medicine, The Second Affiliated Hospital and Yuying Children's Hospital of Wenzhou Medical University, Wenzhou, Zhejiang, China.; 4 Otolaryngology department, The Second Affiliated Hospital and Yuying Children's Hospital of Wenzhou Medical University, Wenzhou, Zhejiang, China.

**Keywords:** Non-small cell lung cancer (NSCLC), postoperative platelet/preoperative platelet ratio (PPR), prognosis, time-dependent ROC

## Abstract

**Purpose:** To determine the prognostic significance of postoperative platelet/preoperative platelet ratio (PPR) in patients with operable non-small cell lung cancer (NSCLC), and assess its prognostic benefit compared to models relying solely preoperative platelet counts (PLT).

**Materials and Methods:** A retrospective analysis of 403 patients who underwent radical resection of NSCLC in our institution from 2013 to 2018 was conducted to assess the prognostic significance of PLT and PPR. Progression-free survival (PFS) and overall survival (OS) were performed by the Kaplan-Meier method. Single-factor and multi-factor COX regression models were used to determine factors that affect long-term outcomes. Time-dependent ROC was used to evaluate the value of PPR in predicting the prognosis.

**Results:** A significant association between high PLT and PPR and poor long-term patient survival outcomes was observed. The median PFS and OS of NSCLC patients with high PLT were 25 months and 29 months, which was significantly shorter than that of patients with low PLT (30 months and 33 months) (both *P* = 0.002). In addition, the median PFS and OS of NSCLC patients with high PPR were 18 months and 26.5 months, which was significantly shorter than that of patients with low PPR (33 months and 35 months) (both *P*<0.001). Univariate and Multivariate analysis using Cox regression model showed that PLT and PPR were independent factors affecting PFS and OS. Time-dependent ROC showed that the predictive capability of PLT and PPR preserved well when they were compared over time following surgery. The AUCs of PLT and PPR to predict 1-year PFS and OS, 3-year PFS and OS, 5-year PFS and OS stabilized between 0.528-0.607. PPR showed significantly higher accuracy than PLT in the prediction of 1-year and 3-year PFS and OS.

**Conclusions:** Elevated PPR is significantly related to the adverse outcomes of patients with NSCLC. PPR can stably predict the long-term prognosis of patients, and can be used as a reliable indicator for evaluating the prognosis of patients with operable NSCLC.

## Introduction

In 2020, approximately 2.2 million of the 19.3 million new cancer cases are lung cancer patients, which led to the second-most commonly diagnosed cancer and the highest mortality rate of cancer among all cancers worldwide 1. About 85% of lung cancers are non-small cell lung cancer (NSCLC). The diagnosis and treatment of lung cancer has made great progress in the past few decades, but the prognosis of lung cancer is still poor due to high recurrence rate and tumor metastasis rate, and the age-standardized 5-year survival rate is only 10.0%-20.0% 2. Clinically, tumor-lymph node-metastasis (TNM) staging system is usually used to evaluate the prognosis of the disease 3, but it cannot accurately predict the clinical prognosis of the disease, especially early cancer 4. Therefore, it is necessary to seek a suitable and effective method to predict the prognosis of patients with NSCLC.

In the past few decades, increasing studies highlighted platelets affect the cancer burden and treatment outcome of cancer patients, and high platelet counts are associated with poor prognosis of tumor patients, including non-small cell lung cancer (NSCLC) 5-7, gastric cancer 8, colorectal cancer 9, ovarian cancer 10, and breast cancer. The underlying reason is that activated platelets induced by tumors play vital roles in tumor growth, tumor cell extravasation, tissue invasion and metastasis of cancer cells 11-15.

However, platelet counts are significantly different between gender, age, and ethnicity 16, and the reference ranges in different regions are quite different. This leads to difficult work in formulating platelet count standards for predicting tumor prognosis and implementing them in clinical practice 6, 17, 18. Furthermore, the progression and recurrence of tumors is a dynamic process, it is difficult to accurately predict the prognosis of patients with the preoperative platelet counts of tumor patients. Hence, postoperative platelet/preoperative platelet ratio (PPR), an index to comprehensively evaluate the dynamic changes of platelets before and after surgery, may be a fine choice in predicting survival outcomes and used to develop a novel prognostic model for patients with NSCLC.

In this study, we verify the correlation between high PLT and poor prognosis in patients with NSCLC, which was consistent with previous research. High PPR was significantly associated with poor long-term patient survival outcomes. And PPR was an independent factor affecting PFS and OS of patients with NSCLC. Time-dependent ROC showed that the predictive capability of PPR preserved well when they were compared over time following surgery. And PPR showed significantly higher accuracy than PLT in the prediction of 1-year and 3-year PFS and OS.

## Materials and Methods

### Patients

This study enrolled patients who underwent radical resection of NSCLC in the Second Affiliated Hospital and Yuying Children's Hospital of Wenzhou Medical from September 2013 to December 2018. All patients were confirmed by pathological examination and immunohistochemistry. Comprehensive clinical, laboratorial, and pathological information was collected. The exclusion criteria are as follows: patients with essential thrombocytosis or long-term use of antiplatelet drugs; patients who were concurrently or previously diagnosed with other organs malignancies or received neoadjuvant therapy; patients were diagnosed with stage IV NSCLC after surgery; and patients who had incomplete data. All studies were conducted following the Declaration of Helsinki.

### Follow-up

After surgery, patients were followed up regularly until death or December 2020 and underwent routine laboratory and imaging tests for evaluation during follow-up. The Progression-free survival (PFS) was calculated from the date of operation to tumor progression or December 2020, the OS was to death or December 2020. The median follow-up time was 28 months (range: 1-88 months). And the lost to follow-up rate was 8.6% (38/441).

### Variables

Study variables, including age, gender, smoking status, pathological TNM staging, Immunohistochemistry and molecular pathology information, were collected. Peripheral blood cell parameters were obtained from laboratory results 3 days before and 2 weeks after surgery. PPR was defined as the ratio of the postoperative platelet count to the preoperative platelet count. The optimal cut-off value for PPR and PLT was 12.1 and 234.5, respectively, which was determined by X-tile software (ver.3.6.1) 19.

### Statistical Analysis

Median and interquartile range (IQR) were used for descriptive statistics of continuous variables, and frequency and proportion were used for categorical variables. The Mann-Whitney U-test and Chi-square test were used to assess the statistical significance between groups. The PFS and OS curves were analyzed by the Kaplan-Meier method. Univariate and multivariate COX risk regression analysis are used to select and determine variables that have independent relationships with recurrence and OS. The time-dependent ROC curve was used to evaluate the ability of variables to predict PFS and OS. SPSS (v. 23.0, IBM, Inc., NY, USA) was used for statistical analyses. The Kaplan-Meier and time dependent receiver operating characteristic (ROC) curves was performed using R (ver. 4.0.3). *P*<0.05 was considered statistically significant.

## Results

### Clinical characteristics of the Patients

**Figure 1:**
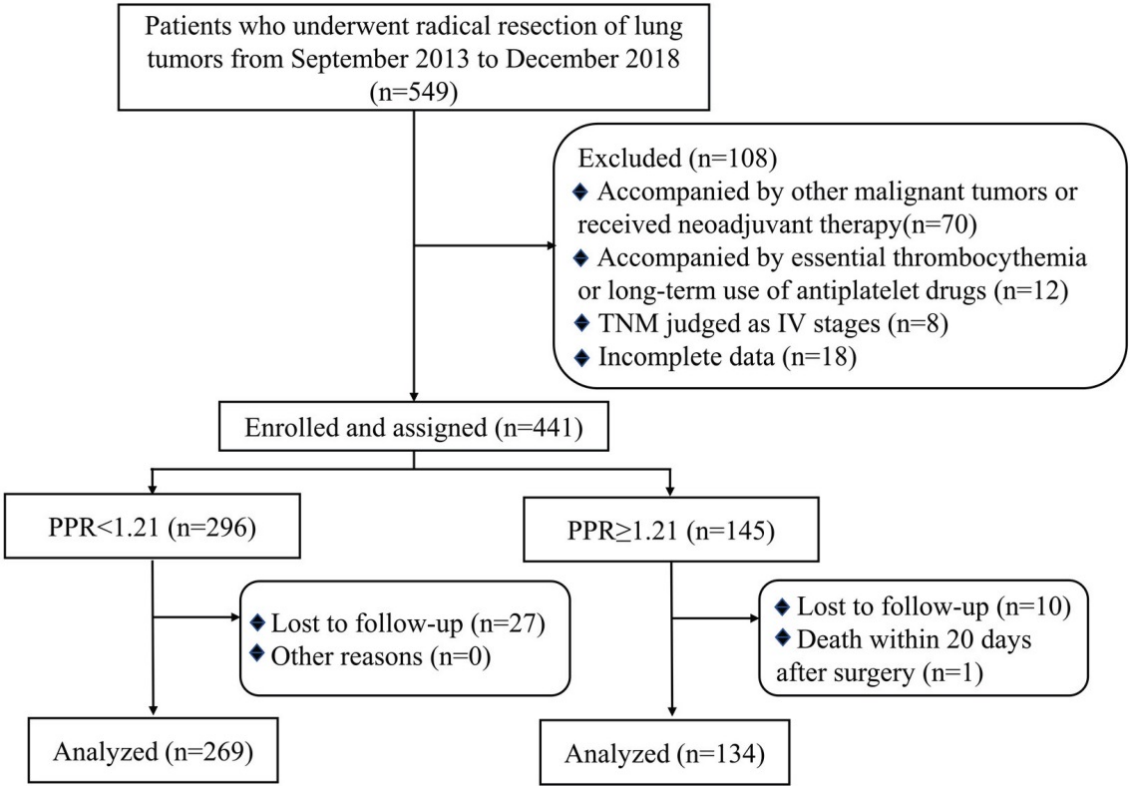
Flow chart of the criteria used to select the patients for inclusion in the present study; **Abbreviation:** TNM, tumor Node Metastasis; PPR, postoperative platelet/preoperative platelet ratio.

During the investigation period, 549 patients were retrospectively analyzed, of which 108 met the exclusion criteria and 38 lost to follow-up (Figure 1). The general characteristics of the 403 patients with NSCLC included in the final analysis were shown in Table 1. 269 patients (66.7%) were classified as the low PPR (<1.21) group, and 134 patients (33.3%) were classified as the high PPR (≥1.21) group. The median age of patients was 62 (53-69) years old. 226 of 403 (56.1%) patients were male and 151 of 403 (37.5%) were smokers. No significant differences were found between the low PPR group and the high PPR group, including gender, age, smoking, microvascular invasion, EGFR mutation, ALK mutation and adjuvant therapy. But the ratio of patients with TNM stage III, tumor > 5cm, and elevated ki67 were higher in the high PPR group, compared with the low PPR group (*P*<0.05).

### Correlation of PLT and PPR with prognosis of patients with NSCLC

**Figure 2:**
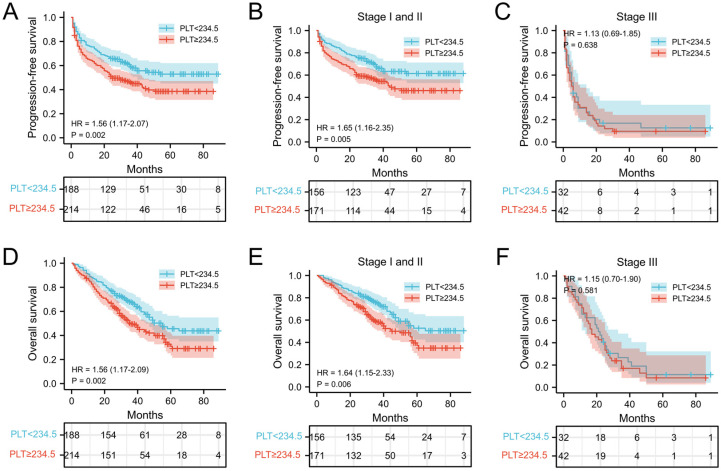
Kaplan-Meier estimates of PFS for overall patients with NSCLC (A), stage I and II patients (B), and stage III patient (C) according to PLT<234.5 and PLT≥234.5; Kaplan-Meier estimates of OS for overall patients with NSCLC (D), stage I and II patients (E), and stage III (F) patient according to PLT<234.5 and PLT≥234.5; **Abbreviation:** PLT, preoperative platelet; HR, hazard ratio.

**Figure 3:**
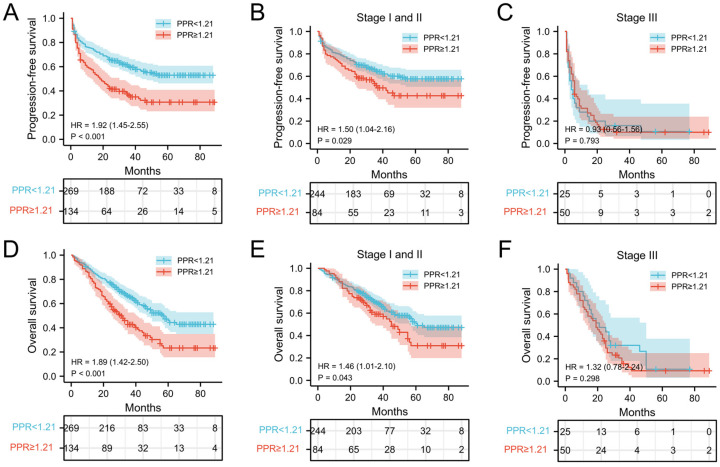
Kaplan-Meier estimates of PFS for overall patients with NSCLC (A), stage I and II patients (B), and stage III patient (C) according to PPR<1.21 and PPR≥1.21; Kaplan-Meier estimates of OS for overall patients with NSCLC (D), stage I and II patients (E), and stage III (F) patient according to PPR<1.21 and PPR≥1.21; **Abbreviation:** PPR, postoperative platelet/preoperative platelet ratio; HR, hazard ratio.

The relationship between PLT, PPR, and the postoperative survival time of patients were performed by survival analysis and the log-rank test. In our cohort, the median PFS and OS of NSCLC patients with PLT < 234.5 ×10^9^/L was 30 months and 33 months, respectively, which was significantly longer than that of patients with PLT ≥ 234.5 ×10^9^/L (25 months and 29 months) (both *P* = 0.002) (Figure 2A and 2D). In addition, the median PFS and OS of NSCLC patients with PPR < 1.21 was 33 months and 35 months, which was significantly longer than that of patients with PPR ≥ 1.21(18 months and 26.5 months) (both* P*<0.001) (Figure 3A and 3D). Next, we evaluated the long-term outcomes according to PLT and PPR based on the tumor stage. Interestingly, the OS and PFS of patients with stage I and II NSCLC were significantly different according to PLT and PPR (Figure 2B, 2E, 3B, 3E). No significant difference of PFS and OS was found between high PLT and PPR with low PLT and PPR in III stage NSCLC patients (*P*=0.638, 0.581, 0.793, 0.298) (Figure 2C, 2F, 3C, 3F).

### Factors affecting long-term outcomes

The univariate COX regression model was used to analyze clinicopathological parameters that could be used to predict clinical prognosis. Gender, age, smoking, tumor size, TNM stage, microvascular invasion, Ki67, adjuvant therapy, PLT, PLT2, and PPR were significant prognostic factors for affecting the patients with NSCLC (Table 2 and 3).

Subsequent multivariate COX regression model shown that tumor size, TNM stage, Microvascular invasion, Ki67, PLT, and PPR were independent risk factors for PFS (Table 2). Age, tumor size, TNM stage, Microvascular invasion, Ki67, PLT, and PPR were independent risk factors for OS (Table 3).

### Prognostic Value of PLT and PPR

**Figure 4:**
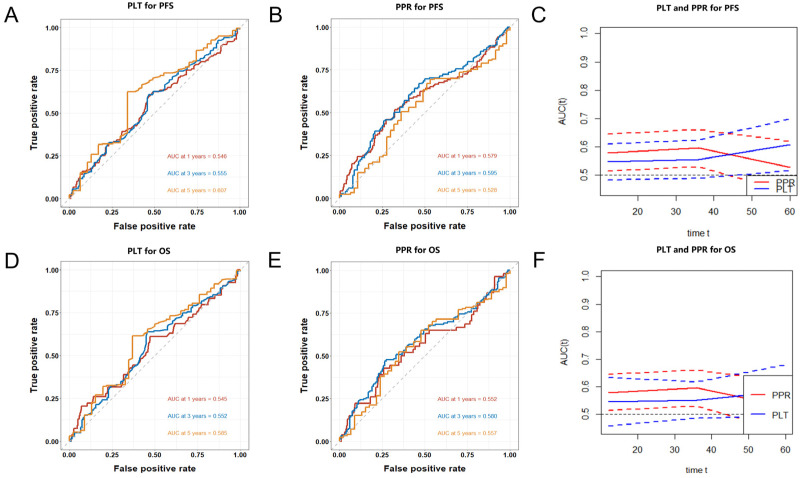
Time-dependent ROC curves of PLT (A) and PPR (B) for the prediction of 1-year, 3-year, 5-year PFS; compare of AUCs of PLT and PPR for the prediction of 1-year, 3-year, 5-year PFS (C); Time-dependent ROC curves of PLT (D) and PPR (E) for the prediction of 1-year, 3-year, 5-year OS; compare of AUCs of PLT and PPR for the prediction of 1-year, 3-year, 5-year OS (F); **Abbreviation:** PPR, postoperative platelet/preoperative platelet ratio; PLT, preoperative platelet; PFS, progression-free survival; OS, overall survival.

Time-dependent ROC curves was used to evaluate the value of PLT and PPR in predict the prognosis. In this cohort, the AUCs of PLT, and PPR to predict 1-year PFS were 0.546 (0.483-0.609) and 0.579 (0.514-0.644), 3-year PFS were 0.555 (0.488-0.622) and 0.595 (0.529-0.660), 5-year PFS were 0.607 (0.515-0.699) and 0.528 (0.437-0.618) (Figure 4A, 4B). The AUCs of PLT, and PPR to predict 1-year OS were 0.545 (0.457-0.633) and 0.552 (0.514-0.644), 3-year OS were 0.552 (0.485-0.618) and 0.580 (0.529-0.660), 5-year OS were 0.585 (0.490-0.679) and 0.557 (0.437-0.618) (Figure 4D, 4E).

The predictive capability of PLT and PPR preserved well when they were compared over time following surgery (Figure 4C, 4F). PPR showed significantly higher accuracy than PLT in the prediction of 1-year and 3-year PFS and OS, but the accuracy in the prediction of 5-year PFS and OS was opposite.

## Discussion

The important role of preoperative PLT in predicting tumor prognosis has been extensively studied 5-10. But it is difficult to be used for personally predicting for the prognosis of patients with cancer in clinic due to the significant difference of platelets between gender, age, and ethnicity. And the progression and recurrence of tumors is a dynamic process, it is difficult to accurately predict the prognosis of patients with the platelet status of tumor patients before surgery. Hence, we proposed a new predictive model, the ratio of postoperative and preoperative platelet counts (PPR). In this study, elevated PPR was associated with poor prognosis of NSCLC patients, especially stage I and II NSCLC patients. PPR was an independent predictor for OS and PFS and the predictive capability of PPR preserved well when they were compared over time following surgery.

Platelets are significantly involved in cancer growth and metastasis and closely related to the prognosis of cancer patients. Tumor cells release ADP 20, 21, matrix metalloproteinases (MMPs) 22, together with thromboxane A2 (TXA2) 23, and activate platelets, resulting in an increased thrombosis and risk of hypercoagulability in cancer patients. Activated platelets induced by tumor cells release a variety of growth factors, including vascular endothelial growth factor (VEGF), platelet-derived growth factor (PDGF), fibroblast growth factor (FGF), and regulate tumor angiogenesis and vascular integrity 24. Activated platelets wrap the circulating tumor cells to form a physical barrier and protect them from the high shear forces in the blood circulation and the attack by leukocytes 14. Platelets release excessive amounts of transforming growth factor-β1 (TGF-β1), which induces the down-regulation of C-type lectin-like NKG2D receptors, resulting in a decrease in the anti-tumor activity of NK cells 25. CLEC-2 on platelets interacts with podoplanin on tumor cells, which induces epithelial-mesenchymal transition (EMT) and enhances tumor cell proliferation and invasion capabilities 26, 27. Hence, not only the increased preoperative platelet count may lead to a poor prognosis for cancer patients, but also the continued increase in postoperative platelet counts may also affect the burden of disease and the treatment effect of cancer patients.

In this study, we verified that elevated preoperative PLT was associated with poor prognosis of patients with NSCLC, especially patients with stage I and II NSCLC, which was consistent with previous studies 5, 6, 28, 29. Importantly, increased PPR was association with shorter PFS and OS of patients with NSCLC, which confirmed that a further increase of postoperative platelet counts in cancer patients often indicates higher risk of recurrence and poor prognostic outcome of patients with NSCLC. According to previous research, even if patients with early-stage cancer successfully undergo radical surgery, a considerable number of patients still exist minimal residual disease (MRD) 30. As an important factor for tumor angiogenesis, tumor cell proliferation, invasion, immune escape and metastasis, platelets may accelerate the progression of MRD in patients undergoing radical cancer treatment. The above phenomenon is more obvious in patients with further increase of platelets after surgery. However, no significant difference of PFS and OS was found between high PLT and PPR with low PLT and PPR in III stage NSCLC patients, which may be due to the insufficient number of patients included in the analysis. The proportion of patients with stage III NSCLC was only 18.6% (75/403) in this cohort and more studies need to be investigated in the future.

Finally, time-dependent ROC further confirmed the value of PPR in predicting the prognosis of patients with NSCLC. The AUCs of PPR to predict 1-year PFS and OS, 3-year PFS and OS, 5-year PFS and OS stabilized between 0.528-0.595. And the predictive value of PPR for PFS and OS of patients with stage I and II NSCLC was higher than that of PLT. The above indicated that the predictive capability of PPR preserved well when they were compared over time following surgery.

At present, it is undeniable that the TNM staging system is still the most distinguished method for the prognosis of NSCLC and the most useful tool for determining the treatment plan for NSCLC patients. However, the TNM staging system still has certain limitations, there are significant differences in the prognosis of tumor patients with the same stage. Therefore, more indicators are needed to comprehensively evaluate the prognosis of patients. PPR is significantly related to the prognosis of NSCLC patients and an independent predictor of the prognosis of operable NSCLC patients. It can further stratify cancer patients of the same stage and provide more individualized prognostic information for cancer patients. In addition, the peripheral blood count is a routine test item for preoperative physical examination and postoperative follow-up. Therefore, PPR is not only economical, but also easy to obtain, continuously monitor, and implement and apply in clinical practice, and is a potential prognostic biomarker.

It is undeniable that our study has several limitations. First, this study is a retrospective analysis in single-institutional, and future prospective, multi-center, large-sample studies are needed to verify the prognostic value of PPR in NSCLC and determine the optimal PPR cut-off value. Second, although we repeatedly review the follow-up information of patients, there are still some patients whose follow-up interval is as long as 1 year or more, which may lead to a certain degree of bias in the PFS of patients.

## Conclusions

Elevated PPR is significantly related to the adverse outcomes of patients with NSCLC, and can stably predict the long-term prognosis of patients, and can be used as a reliable indicator for evaluating the prognosis of patients with operable NSCLC.

## Figures and Tables

**Table 1:** Demographic and Clinicopathological Characteristics of Patients with NSCLC.

Variables	Overall (n=403)	PPR<1.21 (n=269)	PPR≥1.21 (n=134)	*P-value*
**Gender, n (%)**				0.144
Male	226 (56.1%)	144 (53.5%)	82 (61.2%)	
Female	177 (43.9%)	125 (46.5%)	52 (38.8%)	
**Age(years), Median (IQR)**	62 (53-69)	61 (53-67)	63 (54-71)	0.054
**Smoking, n (%)**				0.696
Yes	151 (37.5%)	99 (36.8%)	52 (38.8%)	
No	252 (62.5%)	170 (63.2%)	82 (61.2%)	
**Tumor size**				0.001
≤5cm	356 (88.3%)	248 (92.2%)	108 (80.6%)	
>5cm	47 (11.7%)	21 (7.8%)	26 (19.4%)	
**pT, n (%)**				0.025
≤T2	342 (84.8%)	237 (88.1%)	105 (78.3%)	
T3	43 (10.7%)	24 (8.9%)	19 (14.2%)	
T4	18 (4.5%)	8 (3.0%)	10 (7.5%)	
**pN, n (%)**				<0.001
Negative	286 (71.0%)	222 (82.5%)	64 (47.8%)	
Positive	117 (29.0%)	47 (17.5%)	70 (52.2%)	
**TNM, n (%)**				<0.001
Ⅰ	249 (61.8%)	195 (72.5%)	54 (40.3%)	
Ⅱ	79 (19.6%)	49 (18.2%)	30 (22.4%)	
Ⅲ	75 (18.6%)	25 (9.3%)	50 (37.3%)	
**Microvascular invasion**				0.088
Yes	175 (43.4%)	102 (37.9%)	61 (45.5%)	
No	228 (56.6%)	167 (62.1%)	73 (54.5%)	
**Ki67 (%), Median (IQR)**	15 (5-40)	10 (5-35)	20 (10-50)	0.004
**EGFR mutation, n (%)**				0.878
Yes	53 (13.1%)	37 (13.8%)	16 (11.9%)	
No	33 (8.2%)	22 (8.2%)	11 (8.2%)	
NA	317 (78.7%)	210 (78.0%)	107 (79.9%)	
**ALK mutation, n (%)**				0.787
Yes	5 (1.2%)	4 (1.5%)	1 (0.7%)	
No	81 (20.1%)	55 (20.4%)	26 (19.4%)	
NA	317 (78.7%)	210 (78.1%)	107 (79.9%)	
Adjuvant Therapy*, n (%)				0.074
Yes	137 (34.0%)	83 (30.9%)	54 (40.3%)	
No	266 (66.0%)	186 (69.1%)	80 (59.7%)	
**WBC(x10^9^/L), Median (IQR)**	6.33 (5.36-7.88)	6.39 (5.27-8.14)	6.27 (5.46-7.41)	0.146

**Abbreviation**: PPR, postoperative platelet/preoperative platelet ratio; IQR, interquartile range; TNM, Tumor Node Metastasis; EGFR, epidermal growth factor receptor; ALK, anaplastic Lymphoma kinase. * Adjuvant radiotherapy and/or adjuvant chemotherapy.

**Table 2:** Univariate and multivariate Cox regression analysis for prediction of PFS after RC for NSCLC.

	Univariate analysis		Multivariate analysis	
	Hazard ratio (95%CI)	*P*-value	Hazard ratio (95%CI)	*P*-value
**Gender, n (%)**		0.003		0.939
Male	Ref		Ref	
Female	1.530 (1.151-2.034)		0.986 (0.694-1.401)	
**Age(years)**		0.002		0.045
<60	Ref		Ref	
≥60	0.785 (0.675-0.914)		1.379 (1.008-1.886)	
**Smoking, n (%)**		0.001		0.050
Yes	Ref		Ref	
No	0.794 (0.690-0.914)		1.409 (0.999-1.986)	
**Tumor size, cm**		<0.001		0.024
≤5	Ref		Ref	
>5	1.519 (1.307-1.764)		1.636 (1.069-2.505)	
**TNM, n (%)**		<0.001		<0.001
LG	Ref		Ref	
HG	0.224 (0.165-0.304)		0.493 (0.334-0.726)	
**Microvascular invasion**		<0.001		0.005
Yes	Ref		Ref	
No	0.564 (0.487-0.652)		1.597 (1.151-2.215)	
**Ki67 (%)**		<0.001		<0.001
<15	Ref		Ref	
≥15	0.264 (0.192-0.362)		2.173 (1.530-3.086)	
Adjuvant Therapy*, n		<0.001		0.080
**(%)**				
Yes	Ref		Ref	
No	2.045 (1.544-2.709)		0.760 (0.559-1.034)	
**WBC (x10^9^/L)**		0.492		
<5.775	Ref			
≥5.775	0.890 (0.638-1.241)			
**PLT (x10^9^/L)**		0.001		0.009
<234.5	Ref		Ref	
≥234.5	0.610 (0.458-0.813)		1.565 (1.119-2.188)	
**PLT2 (x10^9^/L)**		<0.001		0.551
<253.5	Ref		Ref	
≥253.5	0.431 (0.323-0.575)		1.120 (0.772-1.624)	
**PPR**		<0.001		0.039
<1.21	Ref		Ref	
≥1.21	0.481 (0.363-0.638)		1.452 (1.019-2.069)	

Abbreviation: CI, confidence interval; HG, high grade; NSCLC, non-small cell lung cancer; TNM, tumor Node Metastasis; WBC, white blood cell; PLT, preoperative platelet; PLT2, postoperative platelet; PPR, postoperative platelet/preoperative platelet ratio; Ref, reference. * Adjuvant radiotherapy and/or adjuvant chemotherapy.

**Table 3:** Univariate and multivariate Cox regression analysis for prediction of OS after RC for NSCLC.

	Univariate analysis		Multivariate analysis	
	Hazard ratio (95%CI)	*P*-value	Hazard ratio (95%CI)	*P*-value
**Gender, n (%)**		0.004		0.930
Male	Ref		Ref	
Female	0.812 (0.705-0.936)		0.985 (0.697-1.391)	
**Age(years)**		0.001		0.002
<60	Ref		Ref	
≥60	0.753 (0.647-0.876)		1.659 (1.211-2.272)	
**Smoking, n (%)**		<0.001		0.015
Yes	Ref		Ref	
No	0.775 (0.674-0.890)		1.528 (1.087-2.148)	
**Tumor size, cm**		<0.001		0.301
≤5	Ref		Ref	
>5	0.597 (0.499-0.714)		1.254 (0.817-1.926)	
**TNM, n (%)**		<0.001		0.003
LG	Ref			
HG	0.283 (0.210-0.382)		Ref	
**Microvascular invasion**		<0.001	0.557 (0.379-0.817)	0.002
Yes	Ref		Ref	
No	0.577 (0.500-0.667)		1.675 (1.205-2.326)	
**Ki67 (%)**		<0.001		<0.001
<15	Ref		Ref	
≥15	0.552 (0.473-0.644)		1.940 (1.383-2.721)	
Adjuvant Therapy*, n		<0.001		0.224
**(%)**				
Yes	Ref		Ref	
No	1.303 (1.133-1.499)		0.824 (0.603-1.126)	
**WBC (x10^9^/L)**		0.300		
<5.775	Ref			
≥5.775	0.915 (0.774-1.082)			
**PLT (x10^9^/L)**		<0.001		<0.001
<234.5	Ref		Ref	
≥234.5	0.764 (0.662-0.882)		1.875 (1,330-2.592)	
**PLT2**		<0.001		0.655
<253.5	Ref		Ref	
≥253.5	0.664 (0.575-0.766)		0.918 (0.631-1.335)	
**PPR**		<0.001		<0.001
<1.21	Ref		Ref	
≥1.21	0.672 (0.584-0.772)		2.046 (1.428-2.932)	

**Abbreviation:** CI, confidence interval; HG, high grade; NSCLC, non-small cell lung cancer; TNM, Tumor Node Metastasis; WBC, white blood cell; PLT, preoperative platelet; PLT2, postoperative platelet; PPR, postoperative platelet/preoperative platelet ratio; Ref, reference. * Adjuvant radiotherapy and/or adjuvant chemotherapy.
